# Self-care and quality of life among men with chronic heart failure

**DOI:** 10.3389/fpubh.2022.942305

**Published:** 2022-07-22

**Authors:** Alicja Wiśnicka, Katarzyna Lomper, Izabella Uchmanowicz

**Affiliations:** Department of Clinical Nursing, Wroclaw Medical University, Wroclaw, Poland

**Keywords:** heart failure, chronic heart failure, self-care, self-care behaviors, quality of life

## Abstract

**Introduction:**

Chronic Heart Failure (CHF) involves a complex regimen of daily self-care behaviors: pharmacological therapy, symptom monitoring and lifestyle modifications. Patients with CHF may have a reduced health related quality of life (HRQoL) due to various physical and emotional symptoms. HRQoL may be improved through the use of self-care interventions.

**Purpose:**

To assess the level of self-care and quality of life among men with chronic heart failure.

**Methods:**

The study was conducted among 80 men diagnosed with CHF (mean age 58 years). The study was cross-sectional. A self-administered questionnaire and analysis of medical records were used to collect baseline sociodemographic and clinical data. Self-care was assessed using the standardized European Heart Failure Self-care Behavior Scale- EHFScBS-9 and quality of life was assessed using the World Health Organization Quality of Life Bref.

**Results:**

The Patients in NYHA class II constituted the vast majority (71.25%), mean LVEF in the study group was 43.5%, and mean disease duration was 3 years. The most common comorbidities were ischemic heart disease (72.5%), hypertension (70%) and diabetes mellitus (60%). The most commonly reported non-pharmacological treatments for NS were fluid restriction (45%), moderate physical activity (42.50%) and daily weight control (41.25%). The EHFSc-9 questionnaire score averaged 50.31 points out of 100 possible (SD = 26.52). The mean score regarding perception of QoL was 2.78 points (SD = 0.91), and 40% of patients indicating poor perception of QoL. The mean score for self-rated Analysis of the results of the individual domains of the WHOQoL BREF questionnaire showed that patients rated their QoL best in the environmental domain (*M* = 13.28; SD = 3.11), then in the social domain (*M* = 12.81; SD = 2.71), and in the psychological domain (*M* = 12.8; SD = 3.2). In contrast, QoL in the physical domain was rated the lowest (*M* = 10.44; SD = 2.85). There was no significant correlation between quality of life and self-care (*p* > 0.05).

**Conclusions:**

Men with CHF have unsatisfactory self-care outcomes and low quality of life scores and are dissatisfied with their health. Strategies to improve selfcare and quality of life in this group are indicated.

## Introduction

Despite advances in the treatment of chronic heart failure (CHF), the disease remains one of the one of the most common causes of hospitalization, death and disability, and affects at least 26 million people worldwide each year (1). The European Society of Cardiology heart failure guidelines indicate that as a result of better treatment of cardiovascular disease, the incidence of heart failure is decreasing, but as a result of an aging population, the aggregate incidence is increasing (2).

In the European countries the incidence of HF correlates positively with age, i.e., 1% in those <55 years of age to >10% in those 70 years and older (2). According to available data, 750,000 people are affected by the disease in Poland and ~6,000 patients will die from HF each year. It is indicated that in the next 10 years the prevalence of CHF will increase significantly due to population aging and the hospitalization rate of patients with CHF in Poland is one of the highest in Europe at 547 per 100,000 inhabitants (3).

CHF is a serious clinical and social problem and represents one of the greatest challenges for the health care system. CHF is a clinical syndrome that consists of symptoms such as dyspnea, edema and fatigue, and symptoms such as elevated jugular venous pressure. Due to its complexity, the disease requires a multifaceted approach. An important part of the therapeutic process of CHF is the undertaking of patient-centered self-care activities. Self-care is based on activities which main goal is to prevent the consequences of CHF by regular medication taking, physical activity adjusted to the patient's abilities, monitoring of disease symptoms or regular check-ups (4). Adequate patient involvement in the care process is essential for effective HF treatment because it allows patients to understand what works for them and to agree on a plan for patient participation in monitoring and treatment (2). As Jaarsma et al. point out, an important part of the therapeutic process in heart failure is the implementation of interventions that foster understanding of the nature of the disease and show patients the importance of the self-care process (5).

Self-care is assumed to have a beneficial effect on the outcome of the therapeutic process among patients with CHF. An adequate level of self-care allows patients to understand the disease, but also to understand the importance of self-management and the treatment process itself. Patients with improved self-care outcomes have a better quality of life (QoL) and lower rates of rehospitalizations and mortality. However, as the CHF treatment guidelines indicate, the quality of life (QoL) of patients is significantly reduced (2). The physical symptoms are often accompanied by depressive and anxiety symptoms, which may additionally contribute to the deterioration of the perceived quality of life. It should also be emphasized that patients with CHF have a worse QoL compared to the general population, but also to patients with other chronic diseases (6). Low QoL in patients with HF is linked to negative clinical outcomes (7). It has been documented that poorer quality of life is associated with rehospitalizations and also increases the risk of death among patients with CHF. Moreover, the impact of QoL on clinical outcomes is as important as the impact of clinical variables such as diabetes mellitus or a history of treatment with angitensin-converting enzyme inhibitors (8). Additionally, it is indicated that men diagnosed with CHF have a better quality of life compared to women and experience less disease symptom burden (9).

In recent years, increasing attention in the health care system has been given to assessing the patient's own perception of illness. The assessment and viewpoint of the patient is increasingly identified as the foundation of quality health care services. A very important aspect of clinical care is the assessment of Patient-reported outcome measures (PROMs) which capture a patient's perception of their own health using standardized questionnaires. The use of PROMs in daily clinical practice supports clinical decision making, helps to prioritize patient care, or stimulates quality improvement in health care services (10).

Therefore, the aim of the study was to assess the level of self-care and quality of life among men with chronic heart failure. We also evaluated correlation between QoL and self-care.

## Materials and methods

### Study settings and participants

The cross-sectional study included a group of 80 men (mean age 58 years) diagnosed with chronic heart failure and hospitalized at the Heart Disease Center, 4th Military Clinical Hospital with Polyclinic SP ZOZ in Wrocław. Data for the study were collected between February 2019 and October 2020. The study was conducted in accordance with the principles of the Declaration of Helsinki. Patients' participation in the study was voluntary and anonymous. The inclusion criteria were: age ≤ 65 years, disease duration ≥ 6 months; NYHA class ≤ III, patient's condition not requiring intensive cardiac care and informed consent. The consented patients were interviewed by the co-author of this study, a trained cardiac nurse.

### Ethical consideration

The study was approved by the independent Bioethics Committee of the Wroclaw Medical University, Poland (no 46/2019). All participants were informed about the purpose of the study, conduct, and the possibility of withdrawal at any stage. The study was conducted in accordance with the requirements of the Declaration of Helsinki.

### Research tools

Basic sociodemographic and clinical data were collected by analyzing the patient's medical records during hospitalization and a self-administered questionnaire interview. The following clinical parameters were obtained from the medical records: BMI, duration of disease, NYHA class, comorbidities, applied pharmacotherapy, LVEF (left ventricular, ejection, fraction), HR, RR values, CRT (cardiac resynchronization therapy), conservative treatment. Sociodemographic data were also obtained during the interview: age, place of residence, marital status, education. After obtaining all data, they were statistically analyzed.

Standardized research tools were used in this study: a quality of life assessment questionnaire: *The World Health Organization Quality of Life Bref* (10, 11) and self-care questionnaire: The *European Heart Failure Self-care Behavior Scale* (12–14).

The WHOQOL-BREF questionnaire—in the Polish adaptation by Wołowicka and Jaracz. It is a standardized tool based on the conceptualization of quality of life as an individual's perception of his/her life position in the context of the culture and value systems in which he/she lives and in relation to his/her goals, standards, expectations, and concerns (11). WHOQOL—BREF contains four domains: physical health, psychological health, social relationships, and environment. Each domain contains between three and eight items. In addition, two general questions provide information on global quality of life (Q1) and health satisfaction (Q2). Each item is based on self-report and rated on a scale of one to five, with higher scores indicating higher quality of life, except for three items that include pain and discomfort, need for treatment, and negative feelings (10).

The study used a Polish adaptation of the European Heart Failure Self-Care Behavior Scale (EHFScB-9) questionnaire to assess HF patients' self-care. The “classic” total score on this questionnaire is a number ranging from 9 to 45, with high scores indicating a low level of self-care. For EHFScB-9, there are no standards regarding how many points can be treated as a high score, and how many as an average. The authors of the tool proposed a simple transformation that changes the scale of the result from 9–45 to 0–100 and reverses it. They called this result “inverted standardized”. In this version, the midpoint is 50, and high scores indicate a high level of self-care (12–14).

### Statistical analysis

Quantitative variables were analyzed by calculating the mean, standard deviation, median, and quartiles. Analysis of qualitative variables was performed by calculating the number and percentage of occurrences of each value. Comparison of the values of quantitative variables in two groups was performed using the Mann-Whitney test. A significance level of 0.05 was assumed in the analysis. Thus, all *p*-values below 0.05 were interpreted as indicating significant relationships. Correlations between variables with non-normal distributions were estimated by Spearman's (rho). The analysis was performed in the program R, version 4.0.3 (15).

## Results

The mean age of the studied group of men was 58 years. The majority were patients residing in a city >300,000 inhabitants, married (65%), with higher education (45%). Sociodemographic data are presented in Table 1.

**Table 1:** Sociodemographic characteristics of the study group (*N* = 80).

**Parameter**		**Total (*****N*** = **80)**
Age (years)	*M* ± SD	55.88 ± 8.24
	Me	58
	Q1–Q3	51.5–62
Place of residence	Village	14 (17.50%)
	City <300,000	28 (35.00%)
	City > 300,000	38 (47.50%)
Marital status	Married	52 (65.00%)
	Alone	12(14.00%)
	Informal relationship	16 (20.00%)
Education	Primary	7 (8.75%)
	Vocational	26 (32.50%)
	Average	11 (13.75%)
	Higher education	36 (45.00%)

*M ± SD, mean ± standard deviation; Me, median; Q, quartiles*.

Analysis of clinical variables showed a mean LVEF in the study group of 43.5%, a mean HR of 66.6 beats per min, a mean SBP of 137 (mmHG), and a mean DBP of 80 (mmHG). Disease duration averaged 3 years, and most patients were classified in NYHA class II (71.25%). The most common comorbidities were hypertension (75%), ischemic heart disease (72.50%), and diabetes mellitus (60%). The majority of patients declared that they did not smoke cigarettes (57.50%), and among smokers, the mean duration of cigarette smoking was 22.5 years. The vast majority of patients declared hospitalizations for HF in the last 6 months (81.25%). ACEI/ARB (81.25%) and B-blockers (48.75%) dominated among the most frequently taken treatment, patients declared taking HF drugs more than once a day (75%). Slightly more than half of the study group (56.25%) declared the use of non-pharmacological methods of HF treatment, of which fluid restriction (45.00%), practicing moderate physical activity (42.50%) and daily weight control (41.25%) were the most common. The use of PDE5 in the last 6 months was declared by 22.5% of patients. Data are presented in Tables 2, 3.

**Table 2:** Clinical characteristics of the study group (*N* = 80).

**Parameter**		**Total (*****N*** = **80)**
LVEF (%)	*M* ± SD	44.54 ± 9.77
	Me	43.5
	Q1–Q3	37–51.75
HR (beats/minute)	M ± SD	67.41 ± 8.46
	Me	66.5
	Q1–Q3	60–72
SBP (mmHg)	*M* ± SD	134.57 ± 16.5
	Me	137
	Q1–Q3	122.25–143.25
DBP (mmHg)	M ± SD	80.44 ± 11.07
	Me	80
	Q1–Q3	75–90
Duration of disease (years)	*M* ± SD	3.5 ± 2.46
	Me	3
	Q1–Q3	1.75–5
NYHA class	NYHA I	4 (5.00%)
	NYHA II	57 (71.25%)
	NYHA III	19 (23.75%)
Comorbidities	Diabetes	48 (60.00%)
	Ischemic heart disease	58 (72.50%)
	Chronic obstructive pulmonary disease	13 (16.25%)
	Chronic kidney disease	27 (33.75%)
	Asthma	6 (7.50%)
	Atrial fibrillation	14 (17.50%)
	Hypertension	60 (75.00%)
Smoking	No	46 (57.50%)
	Yes	34 (42.50%)

*M ± SD, mean ± standard deviation; Me, median; Q, quartiles; LVEF, left ventricular ejection fraction; NYHA, New York Cardiovascular Association; HR, heart rate; SBP, systolic blood pressure; DBP, diastolic blood pressure*.

**Table 3:** Characteristics of the treatment used among the study group (*N* = 80).

**Parameter**		**Total (*****N*** = **80)**
Hospitalizations in the last 6 months	No	15 (18.75%)
	Yes	65 (81.25%)
Number of hospitalizations in the last	0	15 (18.75%)
6 months	Once	27 (33.75%)
	Twice	17 (21.25%)
	Three times and more	21 (26.25%)
Medications taken	Diuretics	42 (52.50%)
	Beta-blockers	39 (48.75%)
	ACEI/ARB	65 (81.25%)
	Aldosterone receptor blockers	25 (31.25%)
	Digoxin	9 (11.25%)
Frequency of heart failure medication taking	Once daily	20 (25.00%)
	More than once a day	60 (75.00%)
Non-pharmacological treatment	No	35 (43.75%)
	Yes	45 (56.25%)
Forms of non-pharmacological treatment	Low-sodium diet	29 (36.25%)
	Weight reduction	26 (32.50%)
	Moderate physical activity	34 (42.50%)
	Limit fluid intake to 1.5–2 l per day	36 (45.00%)
	Daily monitoring of body weight	33 (41.25%)
	Limit fatty foods	32 (40.00%)
Reason for not using non-pharmacological treatment	The recommendations are too complicated for me to follow	3 (3.75%)
	The brochure concerning HF that I received is incomprehensible	5 (6.25%)
	I don't like meals with little salt	5 (6.25%)
	I don't have time to follow recommendations	9 (11.25%)
	I forget about recommendations	9 (11.25%)
	Uwazam ze tabletki sa wystarczajaca forma leczenia	10 (12.50%)

Male patients most often declared a bad (40%) and neither good nor bad (35%) perception of quality of life. In terms of self-perception of health, patients were most often dissatisfied (42.50%) with their health. The data are presented in Table 4.

**Table 4:** Perception of quality of life according to WHOQoL BREF questionnaire and perception of own health according to WHOQoL BREF questionnaire in the study group (*N* = 80).

	***N*** = **80**	**%**
**Perception of quality of life**
Very bad	3	3.75%
Bad	32	40.00%
Neither good nor bad	28	35.00%
Good	14	17.50%
Very good	3	3.75%
**Self-perception of health**
Very dissatisfied	11	13.75%
Dissatisfied	34	42.50%
Neither satisfied nor dissatisfied	21	26.25%
Satisfied	12	15.00%
Very satisfied	2	2.50%

Analyzing the results of the WHOQoL BREF questionnaire, patients rated their quality of life best in the environmental domain (*M* = 13.28), slightly worse in the social domain (*M* = 12.81) and in the psychological domain (*M* = 12.8). On the other hand, quality of life in the physical domain was rated the lowest (*M* = 10.44). The data are presented in Table 5.

**Table 5:** Domains of quality of life according to the WHOQoL BREF questionnaire (*N* = 80).

**WHOQoL BREF**	***N***	***M***	**SD**	**Me**	**Min**	**Max**
Physical domain	80	10.44	2.85	11	5	18
Psychological domain	80	12.8	3.2	13	5	19
Social domain	80	12.81	2.71	13	4	19
Environmental domain	80	13.28	3.11	13	4	19

*M, mean; SD, standard deviation; Min, minimum; Max, maximum; Q, quartiles*.

The EHFSc-9 questionnaire score averaged 50.31 points out of 100 possible (SD = 26.52) and ranged from 8.33 to 97.22 points. The data is presented in Table 6.

**Table 6:** EHFSc-9 Questionnaire results in the study group (*N* = 80).

**EHFSc-9 (points)**
***N***	**Mean**	**SD**	**Median**	**Min**	**Max**
80	50.31	26.52	50	8.33	97.22

Analysis of the relationship between quality of life and self-care showed no statistically significant relationship. We found no statistically significant correlation between quality of life perception (*p* = 0.982), self-perception of health (*p* = 0.27), and physical (*p* = 0.823), psychological (*p* = 0.389), social (*p* = 0.668), and environmental (*p* = 0.695) domains with the EHFSc-9 questionnaire. The data are shown in Table 7.

**Table 7:** The relationship between quality of life and self-care in the study group (*N* = 80).

**WHOQoL BREF**	**EHFSc-9**
	**Spearman correlation coefficient**
Perception of quality of life	*r* = −0.003, *p* = 0.982
Self- perception of health	*r* = 0.125, *p* = 0.27
Physical domain	*r* = −0.025, *p* = 0.823
Psychological domain	*r* = 0.098, *p* = 0.389
Social domain	*r* = −0.049, *p* = 0.668
Environmental domain	*r* = 0.045, *p* = 0.695

## Discussion

Thanks to more effective methods of treatment, the survival of CHF patients has increased, but the course of the disease itself is characterized by frequent exacerbations (16). Exacerbations of the disease can be prevented by using a self-care approach in a group of CHF patients. Patient self-management skills are an important part of heart failure management. It has been shown that patients with CHF who demonstrate high levels of self-care have lower mortality rates and lower rates of rehospitalizations for disease exacerbations (17). Although bothersome symptoms of the disease cause reduced quality of life of patients. Therefore, the main goal of heart failure treatment is to prevent worsening and recurrence of symptoms, improving QoL and prolonging survival time (2).

The importance of self-care in HF is highlighted in the European Society of Cardiology (ESC) guidelines. Self-care in HF should focus on adherence to treatment recommendations, lifestyle modification, monitoring of disease symptoms and the patient's ability to respond to HF exacerbations (2). In our previous studies conducted using the EHFScBS-9 questionnaire in the group of 270 patients with HF we recorded average score of 50.39 points (18). In our another study we conducted among 403 patients, analysis of the EHFSc-9 self-care behavior scale showed that the mean score was 49.55 out of 100 possible points (19). In comparison, da Conceição et al. based on the Self-Care of Heart Failure Index questionnaire, among 116 patients with heart failure (mean age 57.7; SD =11.3), showed an inadequate level of self-care (20). The available literature indicates that men have more difficulty applying selfcare. Mei et al. in a cross-sectional study evaluating self-care behaviors using the Information-Motivation-Behavioral Skills model in 210 CHF patients, found lower levels of self-care in men compared to women (51.4 ± 14.8 in men and 55.6 ± 14.1 in women). Interestingly, associated factors of self-care in men were social support and self-care confidence (21). In addition, Dellafiore et al. in a cross-sectional study of 346 patients with CHF found that men were four times more likely to be at risk for inadequate self-care compared to women (OR 4.596; 95% CI 1.075–19.650) (22). In our study the mean score of EHFScB-9 questionnaire was averaged 50.31 points out of 100 possible, so the results that we obtained are similar to the results in our previous studies (18, 19) but also indicate a low level of self-care in the male group remaining in agreement with the available literature (21, 23). Jaarasma et al. in a meta-analysis of self-care behaviors among 5,964 HF patients reported sub-optimal levels of self-care. Patients reported difficulty in adjusting to behaviors related to physical activity and weight monitoring (2). In our study, <56% of patients declared the use of non-pharmacological methods of HF treatment. The most commonly declared forms of treatment were restriction of fluid intake to 1.5–2.0 L per day, use of moderate physical activity and daily weight control. Among the reasons for non-compliance to non-pharmacological treatment as part of self-care, male respondents most often indicated that receiving pharmacotherapy was a sufficient form of treatment. Seid et al. showed that only 22.3% of patients with heart failure adhered to self-care and non-pharmacologic treatment recommendations, and adherence to self-care recommendations was associated with male gender (AOR = 2.34, 95% CI: 1.18–4.62), high level of knowledge about their own disease (AOR = 2.49, 95% CI: 1.276–4.856), and absence of other chronic diseases (AOR = 2.57, 95% CI: 1.28–5.14) (24).

Non-compliance of HF patients to treatment recommendations, including self-care, is associated with worsening disease symptoms, rehospitalizations, and more deaths. A report published in 2020 indicates that the majority of patients with heart failure in Poland require rehospitalization (25). The obtained results of our own study are in accordance with the published report—as many as 81.25% of patients indicated that they experienced rehospitalization as a result of exacerbation of disease symptoms in the last 6 months. It should be noted that rehospitalizations are associated with worse outcomes in terms of sense of quality of life in the discussed group of patients (8). Additionally, most patients with poor QoL have a worse prognosis and increased severity of heart failure (26).

Based on a cohort study of 319 heart failure patients with reduced ejection fraction (HFrEF) in whom LVEF normalized, Wohlfahrt et al. showed that normalization of left ventricular function was significantly associated with improved quality of life (27). In our study the mean ejection fraction was 43.5%, so the group of men in the study had HF with preserved ejection fraction (EF > 40%) although this result was very close to the borderline of reduced left ventricular ejection fraction (EF <40%). It should be pointed out that in our study the result of assessment of QoL in terms of its perception was not satisfactory—a significant proportion (40%) of the studied men indicated a poor feeling of QoL. In addition, a significant proportion of respondents assessed the perception of their own health as unsatisfactory. Among the reasons for the low values of the QoL questionnaire in this study, one can point to the fact that a significant proportion of the respondents were patients in NYHA class III (23.75%), with a significant decrease in physical activity. This is also reflected in the physical domain of the WHO questionnaire—quality of life in the physical domain was rated the lowest (*M* = 10.44). Analyzing the results of the WHOQoL BREF questionnaire, patients rated their QoL best in the environmental domain, slightly worse in the social domain and in the psychological domain.

Our study strongly suggests that patients with heart failure could also be affected by other factors beyond the physical limitations. Interestingly, we found no statistically significant relationship between quality of life and self-care. Self-care did not affect any domain of QoL in our study. It has previously been shown that low levels of self-care negatively affect feelings of quality of life in a group of patients with heart failure (28). It has also been documented that appropriately managed self-care improves quality of life both inter-subjectively and intra-subjectively (29). However, it should be emphasized that this is a preliminary study and enlarging the research sample may or may not affect further results and observations.

It is indicated that it is necessary to assess the quality of life among patients with CHF because QoL is a multidimensional measure with good correlation with disease severity and it provides independent prognostic information (30). Efforts should also be made to improve self-care among patients with heart failure by creating programs tailored to patients' needs and abilities. In the Polish health care system, efforts have been made to reduce the impact of HF by introducing multispecialty care for patients with heart failure—the KONS program, in which great emphasis is also placed on health education activities (3). It has been proven that health education contributes to a reduction in symptoms and therefore a reduction in rehospitalizations (31). To date, a significant positive effect of motivational interviewing has been shown to improve self-care among patients with CHF (32).

This study provides important information on quality of life and self-care among men, although it has some limitations. This was a single-center study, and a relatively small number of patients were recruited at one center. However, to date, this is the first study assessing selfcare and quality of life in men only.

## Conclusion

Our patients were characterized by inadequate levels of self-care, therefore tailored education is indicated to help the patients achieve optimal self-care values and thus avoid future re-hospitalizations. In our study we also obtained unsatisfactory results in terms of quality of life. Although we found no significant correlation between self-care and QoL, we can conclude that low self-care is associated with poorer QoL. A patient prepared to engage with self-care will have fewer rehospitalizations and a better quality of life.

## Data availability statement

The raw data supporting the conclusions of this article will be made available by the authors, without undue reservation.

## Ethics statement

The studies involving human participants were reviewed and approved by Bioethics Committee of the Wroclaw Medical University, Poland (No. 46/2019). The patients/participants provided their written informed consent to participate in this study.

## Author contributions

AW and IU contributed to conception and design of the study. AW organized the database. KL wrote the first draft of the manuscript. KL, IU, and AW wrote sections of the manuscript. All authors contributed to manuscript revision, read, and approved the submitted version.

## Conflict of interest

The authors declare that the research was conducted in the absence of any commercial or financial relationships that could be construed as a potential conflict of interest.

## Publisher's note

All claims expressed in this article are solely those of the authors and do not necessarily represent those of their affiliated organizations, or those of the publisher, the editors and the reviewers. Any product that may be evaluated in this article, or claim that may be made by its manufacturer, is not guaranteed or endorsed by the publisher.
